# Serpentinization and the Formation of H_2_ and CH_4_ on Celestial Bodies (Planets, Moons, Comets)

**DOI:** 10.1089/ast.2014.1188

**Published:** 2015-07-01

**Authors:** N.G. Holm, C. Oze, O. Mousis, J.H. Waite, A. Guilbert-Lepoutre

**Affiliations:** ^1^ Department of Geological Sciences, Stockholm University, Stockholm, Sweden.; ^2^ Department of Geological Sciences, University of Canterbury, Christchurch, New Zealand.; ^3^ Aix Marseille Université, CNRS, LAM (Laboratoire d'Astrophysique de Marseille) UMR 7326, Marseille, France.; ^4^ Space Science and Engineering Division, Southwest Research Institute, San Antonio, Texas, USA.; ^5^ European Space Agency, Noordwijk, the Netherlands.

## Abstract

Serpentinization involves the hydrolysis and transformation of primary ferromagnesian minerals such as olivine ((Mg,Fe)_2_SiO_4_) and pyroxenes ((Mg,Fe)SiO_3_) to produce H_2_-rich fluids and a variety of secondary minerals over a wide range of environmental conditions. The continual and elevated production of H_2_ is capable of reducing carbon, thus initiating an inorganic pathway to produce organic compounds. The production of H_2_ and H_2_-dependent CH_4_ in serpentinization systems has received significant interdisciplinary interest, especially with regard to the abiotic synthesis of organic compounds and the origins and maintenance of life in Earth's lithosphere and elsewhere in the Universe. Here, serpentinization with an emphasis on the formation of H_2_ and CH_4_ are reviewed within the context of the mineralogy, temperature/pressure, and fluid/gas chemistry present in planetary environments. Whether deep in Earth's interior or in Kuiper Belt Objects in space, serpentinization is a feasible process to invoke as a means of producing astrobiologically indispensable H_2_ capable of reducing carbon to organic compounds. Key Words: Serpentinization—Fischer-Tropsch-type synthesis—Hydrogen formation—Methane formation—Ultramafic rocks. Astrobiology 15, 587–600.

## 1. Introduction

Serpentinization of ultramafic rocks occurring in mid-ocean ridges, forearc systems, and terrestrial ophiolites (*i.e.*, obducted/accretionary oceanic crust) on Earth is a geochemical and water-dependent process that results in a variety of gas and fluid species. Molecular hydrogen (H_2_) is the most influential and relevant species in the abiotic synthesis of organic compounds produced as a consequence of serpentinization due to its ability to reduce carbon (*i.e.*, CO, CO_2_, ${ \rm HCO}_3^ -$, ${\rm CO}_3^{2 -}$) and produce methane (CH_4_) and a wide variety of other organic compounds. Based on serpentinization-related laboratory experiments (Berndt *et al.*, 1996; Seyfried *et al.*, 2007; Klein *et al.*, 2009; Marcaillou *et al.*, 2011; Malvoisin *et al.*, 2012; Velbel *et al.*, 2012; Klein and McCollom, 2013; Etiope and Ionescu, 2014; Lazar *et al.*, 2015), field research (Holm and Charlou, 2001; Charlou *et al.*, 2002; Hosgormez *et al.*, 2008; Proskurowski *et al.*, 2008; Lang *et al.*, 2012a; Okland *et al.*, 2012; Andreani *et al.*, 2013), and theoretical studies (Sleep *et al.*, 2004; Frost and Beard, 2007; Seyfried *et al.*, 2007; Milliken and Rivkin, 2009; Shock and Canovas, 2010; Zolotov, 2014), it is evident that H_2_ production and the concurrent formation of CH_4_ is not a straightforward process, especially when environmental factors, secondary mineral formation, fluid chemistry, mineral chemistry (including rates of Mg-Fe diffusion), fluid flow, carbonate saturation, and time-dependent kinetic processes are considered (*e.g.*, Bach *et al.*, 2006; Evans, 2010; Evans *et al.*, 2013). Regardless of these complicating factors, serpentinization is a common alteration process on Earth and a major pathway for producing H_2_ and CH_4_ over a wide range of geological, environmental, and laboratory conditions, potentially contributing to the origins and early evolution of life (Schrenk *et al.*, 2013). In a broader context, serpentinization and its capability to produce H_2_ and thus initiate the formation of organic compounds, such as CH_4_, is most likely not limited to Earth.

Here, we review several environments in the Solar System where serpentinization and its production of H_2_ and CH_4_ may occur. The topics covered in this review include the principles of the serpentinization process and H_2_ generation (Section 2); Fischer-Tropsch-type (FTT) synthesis and the formation of CH_4_ on Earth and other celestial bodies (Section 3); the synthesis of CH_4_ in astrophysical environments (Section 4); CH_4_ on Mars (Section 5) and Titan/Europa/Enceladus (Section 6); and CH_4_ in comets and Kuiper Belt Objects (KBOs) (Section 7). The intent of this review is to succinctly introduce and cover these specific concepts to provide a useful reference for a wide variety of astrobiological studies. As a result, some tangential facets related to serpentinization may not be fully expounded upon. For example, how serpentinization and its products may be related to abiogenesis and/or the sustainment of life or microbial communities is not fully addressed. Overall, we put forth that serpentinization is, in a true sense, a universal process that leads to the formation of molecular hydrogen and related CH_4_ in a wide variety of environments.

## 2. The Serpentinization Process

**FIG. 1.**
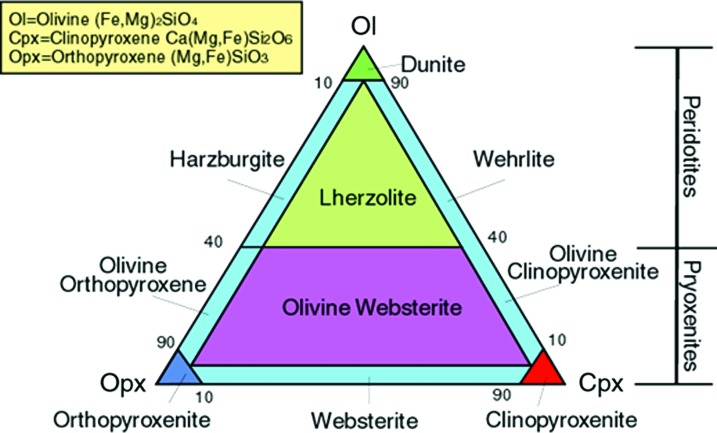
Classification of ultramafic rocks based on Le Maitre (2002). (Color graphics available at www.liebertonline.com/ast)

Serpentinization is primarily associated with ultramafic rocks. An ultramafic rock is defined as a rock containing in total less than about 45 wt % SiO_2_ with elevated Mg and Fe. On Earth, the petrogenesis of ultramafic rocks is related to magmatic and mantle processes. On other celestial bodies, the formation of ultramafic rocks may vary; however, the classification and nature of ultramafic rocks will be the same, as it is based on the rock's chemistry and mineralogy. Additionally, these rocks may be categorized based on their abundance and/or normalized proportions of olivine and pyroxene group minerals (>90% mafic minerals). Ultramafic rocks that contain greater than 40% olivine are referred to as peridotites, whereas rocks with less than 40% olivines are pyroxenites, with further rock subdivisions for each as shown in Fig. 1. Serpentinite is a *catch-all* term for ultramafic rocks that undergo serpentinization, where the extent of serpentinization and/or the abundance of the remaining primary minerals are not directly defined.

**FIG. 2.**
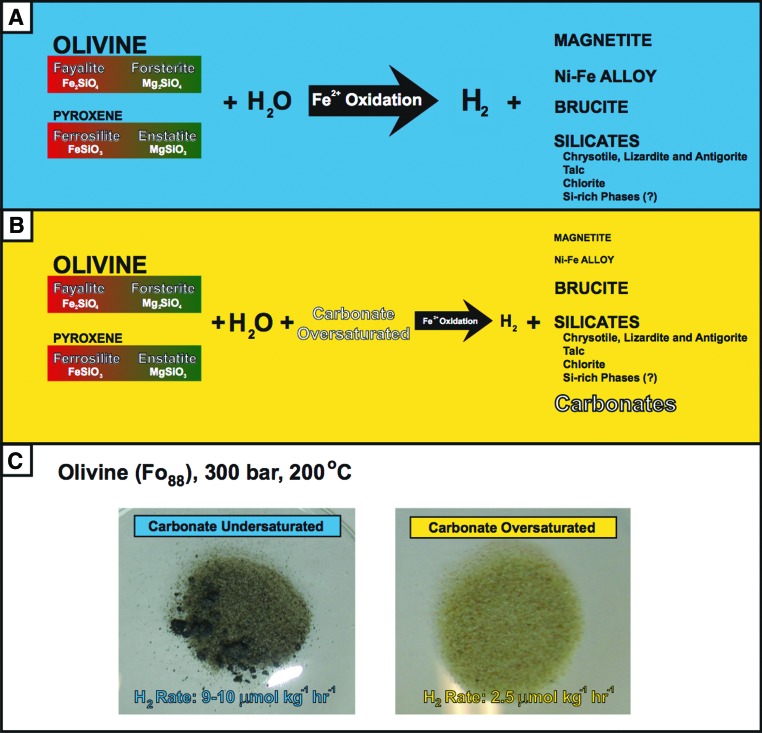
Generic geochemical pathway of (**A**) olivine and pyroxene serpentinization to produce H_2_ and a variety of silicates, oxides, and Ni-Fe alloys and in (**B**) carbonate-oversaturated solutions. The font size reflects the concentration/abundance of minerals/gases/aqueous species involved in the reactions. (**C**) Images of serpentinization experiments from Jones *et al.* (2010) and the resulting solids and H_2_ production rates. The dark color in the carbonate undersaturated experiment is the result of abundant magnetite, whereas very little magnetite is present in the carbonate-oversaturated serpentinization experiment. (Color graphics available at www.liebertonline.com/ast)

The term *serpentinization* generically describes the hydrolysis and transformation of primary ferromagnesian minerals such as olivine ((Mg,Fe)_2_SiO_4_) and pyroxenes ((Mg,Fe)SiO_3_), which may produce serpentine group minerals ((Mg,Fe)_3_Si_2_O_5_(OH)_4_) that include lizardite, chrysotile, and antigorite; magnetite (Fe_3_O_4_); Ni-Fe alloys; talc ((Mg,Fe)_6_(Si_8_O_20_)(OH)_4_); chlorite ((Mg,Fe^2+^,Fe^3+^)_6_AlSi_3_O_10_(OH)_8_); tremolite/actinolite (Ca_2_(Mg,Fe)_5_(Si_8_O_22_)(OH)_2_); and brucite ((Mg,Fe)(OH)_2_), depending on *P-T* parameters. Oxidation of Fe(II) in olivine and pyroxenes leads to the reduction of water and the formation of molecular hydrogen (H_2_) as shown in Fig. 2.

A consequence of serpentinization and H_2_ formation is the increase of the related fluid pH values (typically>9) caused by the consumption of protons and relative increase of free OH^-^ (*e.g.*, Mottl *et al.*, 2003; Okland *et al.*, 2012). Additionally, the production of H_2_ results in fluids that are extremely reducing. As a common reaction pathway, oxidized iron (Fe(III)) is recovered, with Fe(II) to form the mineral magnetite (Fe_3_O_4_). However, upon dissolution of Ni-containing rock-forming minerals, such as olivine, released Ni and Fe can also react to form native metals and alloys under the extreme reducing conditions imposed on the system by serpentinization processes (*e.g.*, Moody, 1976; Sleep *et al.*, 2004; Smirnov *et al.*, 2008). Fe-Ni alloys are known to catalyze the synthesis of CH_4_ and aliphatic hydrocarbons by Fischer-Tropsch synthesis (Nooner *et al.*, 1976; Sleep *et al.*, 2004). In comparison, serpentinization of pyroxene appears to produce less magnetite and more Fe(II)-rich serpentine, decreasing the amount of H_2_ produced (Klein *et al.*, 2013).

In principle, the entire serpentinization process over a wide variety of conditions leads to the formation of serpentine group minerals, magnetite, talc, H_2_, and the magnesium mineral brucite at temperatures less than 315°C (Moody, 1976; McCollom and Bach, 2009; Klein *et al.*, 2014). Key to the formation of H_2_ during serpentinization is the oxidation of Fe(II). The Fe(III) that is formed does not necessarily need to be accommodated in magnetite but can also be hosted in serpentine group minerals during serpentinization under low- to moderate-temperature conditions (<∼200°C) (Klein *et al.*, 2014). Brucite incorporates an increasing amount of Fe(II) with decreasing temperature, thereby decreasing the amount of Fe converted to magnetite (and H_2_O to H_2_) with decreasing temperature below 315°C (Bach *et al.*, 2006; McCollom and Bach, 2009). However, the stability of Fe-rich brucite depends on the activity of H_2_ in the system. If the activity of H_2_ decreases, Fe-bearing brucite will become unstable, and the Fe(II) will be oxidized and reduce water to additional H_2_ (McCollom and Bach, 2009). Therefore, it is possible to register the formation of H_2_ from olivine at relatively low temperatures (<100°C) (Neubeck *et al.*, 2011). Klein *et al.* (2014) demonstrated that H_2_ can be generated as a result of Fe(III) being hosted in serpentine, lending support to the idea that serpentinization processes can generate abundant H_2_ (albeit at longer timescales) within the relatively low-temperature limits of life. This observation agrees with numerous low-temperature serpentinization field observations where H_2_ is present at elevated concentrations (*e.g.*, Morrill *et al.*, 2013).

Serpentinization experiments that involve carbonate-oversaturated fluids at high and low *P-T* conditions have demonstrated that H_2_ concentrations/production rates are decreased relative to those that use carbonate undersaturated fluids (Fig. 2B) (Jones *et al.*, 2010; Klein and McCollom, 2013; Neubeck *et al.*, 2014). Dihydrogen production may be limited due to decreased Fe(II) oxidation and the fast and favorable incorporation of Fe(II) into carbonates, silicates, and metal hydroxides. Most notably, magnetite formation is nearly suppressed in carbonate-oversaturated solutions (Fig. 2C). Additionally, the rate of increasing pH due to H_2_ production serves as a supplemental factor further forcing carbonate formation (Neubeck *et al.*, 2014). The decrease in H_2_ production as well as carbon being directed toward carbonate formation will reduce concurrent serpentinization-related CH_4_ production. Serpentinization experiments in which isotopically tagged carbonate was used resulted in carbonate-oversaturated fluids, and it was concluded that CH_4_ production was limited via serpentinization and CH_4_ was primarily released from olivine (McCollom and Seewald, 2001). However, the production of H_2_ and CH_4_ may have been influenced by the carbonate isotopic tracer (meant to track CH_4_ production) that forced carbonate oversaturation. As carbonate saturation is continually being modified in hydrothermal/serpentinization systems, especially those that involve seawater, H_2_ and CH_4_ production is a dynamic process capable of continuously and rapidly modifying chemical pathways and H_2_ and CH_4_ concentrations.

As discussed above, the serpentinization process does not necessarily produce organic compounds, such as CH_4_, by itself but needs to be combined with other geochemical mechanisms such as FTT processes for that purpose. The production of H_2_ is an essential (indirect or first) step for advancing the abiotic synthesis of organic compounds (Holm and Andersson, 1998). Reactions between single carbon compounds in the C–H–O system are limited to the formation of CO_2_, CO, formic acid (HCOOH), formaldehyde (CH_2_O), methanol (CH_3_OH), and CH_4_ (Seewald *et al.*, 2006). CO is of particular interest because it represents a key reactant during the abiotic synthesis of reduced carbon compounds via FTT processes. Peridotite-hosted hydrothermal systems characterized by high H_2_ abundance and temperatures near 350°C contain substantially higher CO concentrations than basalt-hosted systems, which may enhance abiotic synthesis of longer-chain carbon (Charlou *et al.*, 2002). However, it should be noted that recent studies have demonstrated that H_2_ may not be necessary in serpentinization systems to produce organic compounds such as CH_4_. For example, Suda *et al.* (2014) proposed an alternative pathway for the direct abiotic CH_4_ production from H_2_O (*i.e.*, bypassing mediation by H_2_ reacting with a carbon source) in a serpentinite-hosted hydrothermal system. However, Whiticar and Etiope (2014) put forth that their direct abiotic pathway is compatible with FTT processes and consistent with other serpentinization sites.

**FIG. 3.**
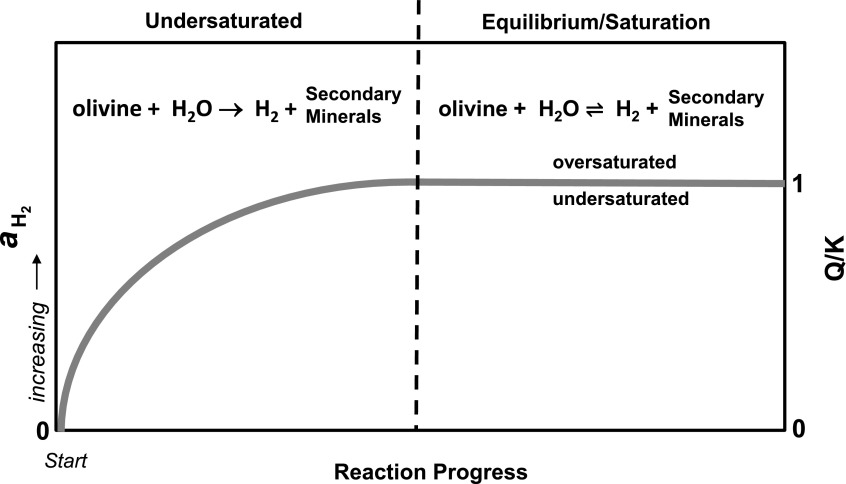
An idealized graphical representation is shown illustrating serpentinization and H_2_ production with regard to saturation/equilibrium and the activity (*a*) of H_2_ (left *y* axis) versus reaction progress (*x* axis). The saturation index [the reaction quotient (*Q*) divided by the equilibrium constant (*K*)] is shown on the right *y* axis. Note that the H_2_ concentration increases (directional arrow) with respect to reaction progress until equilibrium/saturation is achieved (equilibrium arrows). A key point is that H_2_ production will occur as long as the H_2_ concentrations are undersaturated (*Q/K*<1) with respect to equilibrium H_2_. Arguably, serpentinization systems are dominantly undersaturated with respect to equilibrium H_2_ (*i.e.*, at disequilibrium).

Related to mineral composition, the proportion of Fe and Mg in olivine and pyroxene plays a central role with regard to mineral stability and the extent to which H_2_ may be produced. Generally, Fe-rich primary minerals such as the Fe-rich olivine end-member fayalite (Fe_2_SiO_4_) provide a greater opportunity for Fe(II) to oxidize and ultimately form H_2_. Oze and Sharma (2007) assessed a series of serpentinization reactions focusing on Mg and Fe in olivine to determine the Gibbs free energy of reaction and assess H_2_ production in context to each reaction. They demonstrated that serpentinization reactions involving mantle olivine (forsteritic olivine), which is Mg-rich with a relatively smaller proportion of Fe, are thermodynamically favorable (defined as Δ*G*_R_<0), demonstrating higher yields of H_2_ (*i.e.*, H_2_ production) compared to the stoichiometry of the reaction. Conversely, reactions with Fe-rich olivine (<Fo_50_) are generally thermodynamically unfavorable (defined as Δ*G*_R_>0), where H_2_ yields are less than the stoichiometry of the reaction (*i.e.*, H_2_ is consumed or not produced). It should be noted that these interpretations are strictly limited to assessing the reaction and not the system with respect to the degree of H_2_ saturation. An important point is that even a thermodynamically unfavorable serpentinization reaction, such as the Fe-olivine undergoing serpentinization, will have an equilibrium concentration of H_2_. Serpentinization reactions undersaturated with respect to equilibrium H_2_ will produce H_2_ as shown graphically in Fig. 3. Klein *et al.* (2013) expanded on the thermodynamics of Mg and Fe in a wide variety of minerals that may undergo serpentinization and assessed equilibrium H_2_ concentrations using a similar methodology as well as affinity calculations, which reiterate that H_2_-undersaturated systems will produce H_2_. Despite its utility, thermodynamic/equilibrium modeling of H_2_ production as shown by Oze and Sharma (2007), Klein *et al.* (2013), and Sleep *et al.* (2004) does not address the complexity or time-dependent factors and transitory phases capable of modulating H_2_ production in real systems well before equilibrium is achieved (*i.e.*, the left side of Fig. 3). In essence, serpentinization and H_2_ production is a disequilibrium process (as shown and stated in numerous studies) where a wide variety of factors (reaction kinetics, surface reactions, time, fluid flow, etc.) are constantly modifying H_2_ production and H_2_ present in related fluids (Schulte *et al.*, 2006).

Serpentinization is most efficient in fractured rock that allows fast percolation of water (*e.g.*, Moody, 1976; Kelemen and Hirth, 2012). Therefore, serpentinites from oceanic areas are normally associated with fracture zones and trenches (Christensen, 1972). On a global scale on Earth, serpentinization of mantle and deep, hot crustal rocks appears to be a significant process, especially in crustal development in zones of relatively low magmatic supply, like the Mid-Atlantic Ridge and the Gakkel Ridge of the Arctic Ocean. Serpentinization is a fast process as seen on a geological timescale, yet it is not self-terminating since new mineral surfaces are constantly being exposed (Malvoisin *et al.*, 2012). If we assume that H_2_ production is a tracer for this type of weathering/alteration, then serpentinization is a fast process at both low and high temperatures. It is, however, difficult to give precise numbers on the H_2_ production relative to different temperatures due to the complexity of the systems and processes.

## 3. Fischer-Tropsch-Type Synthesis

Fischer-Tropsch-type reactions are unique among abiotic organic processes in providing a source of linear molecules. This is important in a prebiotic context since, for instance, linear fatty acids are essential for the formation of the bilayer membranes that are present in cell walls (Ferris, 1992). The generation of organic compounds by FTT reactions is possible if H_2_ is first generated (Holm and Neubeck, 2009). Fischer-Tropsch-type synthesis broadly refers to the consumption of CO and/or CO_2_ and the production of CH_4_ and longer-chain hydrocarbons as shown in the reactions below:

\begin{align*}( 2n + 1 ) { \rm H}_2 + n{ \rm CO} \rightarrow {
\rm C}_n{ \rm H}_{ ( 2n + 2 ) } + n{ \rm H}_2{ \rm O} \quad\quad {
\rm Reaction \ 1} \end{align*}

\begin{align*}{ \rm CO}_{2 ( { \rm aq} ) }  + [ 2 + ( m / 2n ) ]
{ \rm H}_2 = ( 1 / n ) { \rm C}_n { \rm H}_m + 2{ \rm H}_2{ \rm O}
\quad\quad { \rm Reaction \ 2} \end{align*}

Methane formed from the direct reduction of CO_2_ with aid from a catalyst is a specific FTT reaction referred to as the Sabatier (methanation) process:

\begin{align*}{ \rm CO}_2 + 4{ \rm H}_2 = { \rm CH}_4 + 2{ \rm
H}_2{ \rm O} \quad\quad { \rm Reaction \ 3} \end{align*}

or CH_4_ may form via the two-step reverse water-gas shift reaction:

\begin{align*}{ \rm CO}_2 + { \rm H}_2 = { \rm CO} + { \rm H}_2{
\rm O} \quad\quad { \rm Reaction\ 4} \end{align*}

\begin{align*}{ \rm CO} + 3{ \rm H}_2 = { \rm CH}_4 + { \rm H}_2{
\rm O} \quad\quad { \rm Reaction\ 5} \end{align*}

Commercial FTT reactions are designed to primarily produce linear hydrocarbons, alcohols, and fatty acids from CO and H_2_, but it is also possible to prepare substances like amino acids and heterocyclic nitrogen compounds with limited chain branching by using the FTT reaction of H_2_, CO, and NH_3_ (Hayatsu and Anders, 1981). It was difficult to imagine reliable prebiotic mechanisms for the synthesis of linear fatty acids until deep-sea hydrothermal environments in the lithosphere were discovered on Earth. Earlier, no likely environments were known in which both high temperatures and pressures required for FTT reactions occurred simultaneously with redox conditions at which the synthesized organic molecules are stable (Holm *et al.*, 2006).

Standard FTT reactions are usually performed with catalysts of native metals or alloys of them (Fe, Ni, Co, Ru) or their oxides (Anderson, 1956; Falbe, 1980; Steynberg and Dry, 2004), for example, the Fe(II)-Fe(III) oxide mineral magnetite (Fe_3_O_4_). Commercial FTT reactions have been optimized for the synthesis of hydrocarbons from CO and H_2_ via the water-gas shift reaction. Hayatsu and Anders (1981) extended the FTT reaction to biomolecules by adding NH_3_ to the mixture of gaseous reactants so that nitrogen-containing molecules are produced.

Fischer-Tropsch-type reactions obviously proceed in hydrothermal systems (Holm and Charlou, 2001; Konn *et al.*, 2009) as a result of the reactants being heated at high temperature and pressure in the presence of minerals. The CO needed may come from degradation of, for instance, formaldehyde that can be formed via hydroxymethylene from elemental carbon in the presence of water (Flanagan *et al.*, 1992; Seewald *et al.*, 2006). The H_2_ is most easily formed by the serpentinization of ultramafic rocks. It has been claimed that FTT reactions cannot be involved in the synthesis of organic compounds in hydrothermal systems because of the inhibition of catalysts by both H_2_O and H_2_S (Miller and Bada, 1988). This may be true for native iron as a catalyst (Steynberg and Dry, 2004) but not necessarily for all other possible catalysts. The extent of H_2_S poisoning depends on the ratio of H_2_S to catalytic sites on the metal. The Fe catalyst will continue to function until most of the catalytic sites have H_2_S bound to them. However, in ultramafic off-axis hydrothermal systems of the Lost City type, the concentration of H_2_S is very low (Kelley *et al.*, 2001), so little poisoning will probably occur in relation to the rate of the generation of new mineral surfaces. In addition, some metals other than Fe do not seem to be inhibited by H_2_S. Molybdenum sulfide, nickel sulfide, and tungsten sulfide have all been shown to be active as FTT catalysts (Storch *et al.*, 1951; Asinger, 1968). Recently, Etiope and Ionescu (2014) determined that Ru is an effective catalyst for CH_4_ production at room temperature (20–25°C), offering important implications for Ru-bearing chromite within low-temperature serpentinization systems.

Water is known to be a reversible inhibitor of the reduction of nitrogen to ammonia on Fe catalysts (Storch *et al.*, 1951; Anderson, 1956). However, this is not to say that H_2_O is an inhibitor of all FTT reactions. What is not clear is whether water in the supercritical state binds to the catalysts strongly enough to inhibit the binding and subsequent conversion of N_2_ and CO to organic carbon- and nitrogen-containing molecules. In many cases FTT reactions may even produce water in parallel to organic compounds. One effect of water in some reactions is that the formation of long carbon chains is increased relative to short chains (Storsæter *et al.*, 2005).

Fischer-Tropsch-type reactions apparently do not necessarily require metal or metal oxide catalysts but can also proceed on other mineral surfaces. There are several reports of efficient syntheses proceeding on silica (Asinger, 1968), smectite (montmorillonite), and smectite-alumina mixtures (Yoshino *et al.*, 1971; Anders *et al.*, 1974). This is of particular interest from a marine geochemical point of view since smectites and silica are formed during weathering (palagonitization, serpentinization) of basalts and ultramafic rocks of oceanic basement (da Silva and Holm, 2014).

## 4. Synthesis of Methane in Astrophysical Environments

After hydrogen and helium, the three most abundant elements in the Universe are carbon, nitrogen, and oxygen. Fischer-Tropsch-type catalysis, which converts CO or CO_2_ and H_2_ into CH_4_ and other carbon compounds on the surface of, for instance, transition metals such as Fe and Ni, can then be supposed to play important roles in various astrophysical environments. In addition to producing hydrocarbons, Fischer-Tropsch catalysis also forms water, and this mechanism has been proposed as a chemical route to produce this molecule in the circumstellar envelopes around carbon-rich asymptotic giant branch stars (Kress, 1997; Willacy, 2004). The abundance of water produced via FTT reactions in these environments is found to be consistent with the observations if only a few percent of the available iron exists as metallic Fe or Fe-Ni alloy (Willacy, 2004). Moreover, it has been proposed that FTT reactions could explain the origin of meteoritic organics (Studier *et al.*, 1968; Anders *et al.*, 1973; Hayatsu and Anders, 1981; Bradley *et al.*, 1984). However, this possibility is still debated because experimental studies have shown that the isotopic fractionation patterns found in meteorites do not match those produced via FTT reactions (Cronin and Pizzarello, 1990). Meanwhile, other studies have suggested that FTT reactions were too slow in the protosolar nebula to reduce more than about 1% of CO present over the entire lifetime of the disk (Lewis and Prinn, 1980; Prinn and Fegley, 1981). On the other hand, more recent models of the protosolar nebula provide suitable conditions for synthesis of organic compounds from CO and H_2_ (Kress and Tielens, 2001). These models indicate that FTT reactions could have been efficient in a narrow region of the nebula that coincides with the present position of the Main Belt. More recently, laboratory experiments that determine CH_4_ reaction rates under a hydrogen-dominated gas phase and at low-pressure conditions allowed Sekine *et al.* (2005) to study the ranges of temperatures and pressures where FTT reactions can take place in the saturnian subnebula. They concluded that CH_4_-rich satellitesimals could have formed in the catalytically active region of the subnebula and thus may have played an important role in the origin of Titan's atmosphere. In contrast, investigations led by Mousis *et al.* (2006) confirmed the likely existence of a catalytically active region in the subnebula but estimated that it has no influence on the composition of the forming satellitesimals, because the produced CH_4_ is shown to be accreted by Saturn prior to its trapping in the satellites' building blocks.

Fischer-Tropsch-type catalysis has also been invoked to play an important role in asteroidal/cometary impacts on Earth and bodies of the Solar System (Anders *et al.*, 1973; Gerasimov *et al.*, 2000; Sekine *et al.*, 2003, 2011; Kress and McKay, 2004; Ishimaru *et al.*, 2011). The impactor and a part of the target that are vaporized upon impact would result in the formation of a vapor cloud. Fischer-Tropsch-type reactions leading to the formation of CH_4_ may then occur on the surfaces of dust condensates in the expanding impact vapor cloud (Sekine *et al.*, 2006). By calculating the global CH_4_ production via FTT catalysis on the surface of reentering condensates by iron meteorite impacts on early Earth, Sekine *et al.* (2003) found that the amount of produced CH_4_ may play an important role not only in the atmospheric evolution as a greenhouse effect gas but also in the origin of life. Shock heating on primordial Titan has also been proposed to explain its current atmosphere, which is dominated by N_2_ and CH_4_ (Ishimaru *et al.*, 2011; Sekine *et al.*, 2011). The atmospheric CH_4_ of Titan mainly originates from the protosolar nebula and was trapped in the satellite's building blocks at their condensation epoch in Saturn's feeding zone (Mousis *et al.*, 2009). However, it is possible that a fraction of the existing CH_4_ was produced via FTT reactions that occurred during the entry of impactors in Titan's proto-atmosphere (Ishimaru *et al.*, 2011).

## 5. Mars—Methane or Not?

Recent spacecraft-based and Earth ground-based studies have reported seasonally and latitudinally variable CH_4_ values between 10 and 60 ppbv in the martian atmosphere (Formisano *et al.*, 2004; Krasnopolsky *et al.*, 2004; Geminale *et al.*, 2008, 2011; Mumma *et al.*, 2009; Fonti and Marzo, 2010). In a review of these studies, Zahnle *et al.* (2011) put forth the conclusion that these observations and analyses can only provide an upper limit of 3 ppbv. In 2013, the Curiosity rover with its martian ground-based evaluation of the CH_4_ in the martian atmosphere reported no detection of CH_4_ with a measured value of 0.18±0.67 ppbv and an upper limit of only 1.3 ppbv (Webster *et al.*, 2013), well below all previously reported values. While this reduces the possibility of a wide variety of CH_4_ sources including methanogenic microorganisms as a major contributor to the atmospheric chemistry of Mars, it does not negate the possibility that CH_4_ is being produced in the subsurface at concentrations that will not affect atmospheric chemistry and/or be destroyed by oxidizers such as perchlorate (1–4 wt %) identified on the martian surface (Davila *et al.*, 2013).

Potential sources that have been proposed to release CH_4_ into the martian atmosphere include methanogenic microbes, organic material decomposition, CH_4_ clathrates in the subsurface, volcanism, mantle plumes, comet/meteor impacts, atmospheric interactions, and hydrothermal activity (*e.g.*, Kress and McKay, 2004; Oze and Sharma, 2005; Tung *et al.*, 2005; Prieto-Ballesteros *et al.*, 2006; Ryan *et al.*, 2006; Chastain and Chevrier, 2007; Craddock and Greeley, 2009; Thomas *et al.*, 2009; Brown *et al.*, 2010; Ehlmann *et al.*, 2010; Shkrob *et al.*, 2010; Etiope *et al.*, 2013). None of these appear to be sources of CH_4_ to the atmosphere on their own. However, serpentinization continues to be a viable geochemical route that leads to the formation of H_2_ and, indirectly, to CH_4_ on Mars, perhaps coupled to processes like methanogenic autotrophy and/or clathrate formation. Ultramafic and serpentinized rocks have been identified on the surface of Mars, including locations such as Nili Fossae (Brown *et al.*, 2010; Ehlmann *et al.*, 2010; Viviano *et al.*, 2013). If liquid water (which has been identified on the surface) is present at depth and it interacts with ultramafic material, H_2_ and CH_4_ will be produced (Jakosky and Haberle, 1992). If serpentinization fluids/gases are progressing from a deep to shallow/surface environment, H_2_ and CH_4_ will be quickly dispersed upon reaching the surface and, therefore, would only be detectable via direct monitoring and analyzing soil/regolith/vent gas flux and concentrations in a martian ultramafic environment (Etiope and Sherwood Lollar, 2013). This has yet to be completed. It is questionable why this has not been a major objective, since serpentinization environments with H_2_ and CH_4_ provide a very plausible scenario/locality to harbor and sustain life as well as potentially being a source of energy/fuel for future human habitation. Additionally, low H_2_/CH_4_ values (<46) assessed at the surface in a serpentinization environment may indicate that life is present at depth provided that the complexity of the system and CH_4_ production is governed by the relative kinetics of H_2_ and CH_4_ formation (Lang *et al.*, 2012b, Oze *et al.*, 2012a, 2012b).

## 6. Titan, Europa, Enceladus

The outer Solar System lies beyond the “snow line.” In this region beyond the present-day orbit of Jupiter, volatiles were frozen out, trapped in clathrates and crystalline ice, and mixed with chondritic materials, forming comets and planetesimals (Mousis *et al.*, 2009). Since carbonaceous chondrites represent the most primitive class of chondritic materials, it is generally assumed that they are most representative of the chondritic materials that were gravitationally trapped in the icy materials forming the jovian and saturnian systems. Within the carbonaceous chondrite group, the CI chondrites, which contain the full complement of volatile elements at solar abundance levels, are the class most frequently associated with outer planet satellite formation (see for example, Shock and McKinnon, 1993; Zolotov, 2012). In fact, Kargel *et al.* (2000) pointed out that the density of Europa is the same as the materials that form CI chondrites and that the earlier dehydration of the minerals that formed Europa supplied the water and ice we see in its outer layers today. This leads us to the question of evolution of the original materials through aqueous and thermal alteration and the timing of this thermal and aqueous alteration. As for now, we have no definitive measurements of materials from these satellites to answer this important question.

Once the initial materials coalesced to form the nascent satellite, thermal alteration driven by accretional heating, radioactive decay, and tidal forcing leads to gravitational differentiation and further aqueous alteration of the minerals. Ferromagnesian silicate rocks (*i.e.*, peridotites) prevalent in Earth's mantle may be formed depending on the timing of formation relative to the body's ability to dissipate heat (Schubert *et al.*, 1986; McKinnon and Zolensky, 2003). Furthermore, if the body in its evolution is warmed up enough for liquid water to exist in its interior, cooling eventually takes over, and through the process of serpentinization the perioditic assemblages from a nascent water-deficient high-temperature state re-equilibrate to the water-saturated low-temperature state that characterizes planetary aqueous environments (Vance *et al.*, 2007). Furthermore, Vance *et al.* (2007) added that the rate of serpentinization reactions is controlled by the pressure, temperature, fluid oxidation state and pH, the rate of exposure of fluid access to the serpentinizable rock, and the Fe/Mg ratio of the peridotite material, all of which potentially play a role in determining the longevity of the thermal system. The major factor in small icy satellites is likely the access of the fluid to the rock, which can slow down the kinetic reaction rates by factors of up to one million. This re-equilibration may lead to the production of H_2_, CH_4_, and heat with important implications for astrobiology.

Kargel *et al.* (2000) argued that the thermal alteration processes on Europa not only modified the mineral oxidative balance of the materials but were intense enough to almost fully dehydrate the minerals, causing global gravitational differentiation of the interior ocean and seas. Indeed, due to the melting point and boiling point of water, aqueous alteration and thermal alteration are intimately related. Even in the case of the small body Enceladus, Malamud and Prialnik (2013) argued that extensive short-lived radioisotope decay coupled with runaway serpentinization can lead to a fully differentiated rocky inner core, topped by a thin icy crust. On the other hand, they argued that long-term radioisotope heating in the presence of an NH_3_ solution can result in long-term serpentinization confined to its inner core. This early mineral alteration process forms the basis of the interpretation by Glein *et al.* (2007) of the state of the internal sea at Enceladus interpreted through the gas composition of the plume.

Observations of volatiles released from an interior ocean through volcanic or plume venting can be used to determine the state of past or present hydrothermal processing. Volatiles that source from the ocean should indicate the degree of chemical processing of ocean constituents. Depending on their ratios of N_2_ to NH_3_, ^14^N/^15^N, and CO_2_/CO/CH_4_, as well as H and C isotopic ratios, they may represent an equilibrium, a hydrothermally processed assemblage, or a disequilibrium, cometlike assemblage (Bradley and Summons, 2010). If the ratios appear consistent with thermochemical processing, then constraints on the temperature and oxidation state (fugacity) should be possible. Persistence of reactive carbon-bearing species—CO and especially HCN—on the other hand would point to a more primitive, chemically unprocessed volatile assemblage. In terms of *habitability*, oxidation states more reducing than the fayalite-magnetite-quartz fugacity buffer are consistent with the preservation, if not abiotic synthesis, of more complex organic compounds.

Ongoing hydration (technically hydroxylation) of mafic and especially ultramafic silicate minerals (olivines and pyroxenes) can act as a source of H_2_, which can fuel microbial ecosystems. Evidence of hydrothermal activity also implies a primary source of energy, as well as a likely mechanism to cycle biogenic elements in and out of the ocean. Sputtering of surface ice will yield H_2_O molecules and molecular fragments, but excess H_2_ (and associated isotopes) could indicate ongoing hydrothermal activity (*e.g.*, serpentinization or cracking of organics).

Measurements of the H and C isotopic ratios of carbon-bearing compounds can help distinguish the lineage of carbon chemistry (biotic or abiotic), provided the isotopic composition of the primary “feedstock” can be measured as well (presumably H_2_O and CO_2_): (1) Fischer-Tropsch-type (classic abiotic example), (2) cometary (primordial), and (3) biological.

However, the effect of processes such as solubility and phase changes in the interior sea or ocean must be properly taken into account in the interpretation of the plume gases. Both solubility and clathrate hydrate formation can modify the dissolved gas content of ice-covered lakes on Earth (Mousis *et al.*, 2013) and are likely of importance in icy satellites bearing interior oceans or seas. Furthermore, the clathrates that are formed may float to the surface of the sea or ocean, where they may be destabilized preferentially and release their gases into the plumes.

## 7. Comets and Kuiper Belt Objects

Comets and their parent bodies, such as Transneptunian Objects (Kuiper Belt Objects—KBOs), accreted from a mixture of volatile ices, carbonaceous matter, and rocks in the coldest regions of the protosolar nebula. These objects are usually believed to be very primitive in nature, having preserved a primordial structure and composition. However, the rocky material contained in comets includes radioactive isotopes, whose decay can provide an important source of heat, possibly significantly altering the internal structure of these icy objects after their formation. There is a general agreement that short-lived radioactive isotopes like ^26^Al and ^60^Fe could have played a major role during the early evolution of both comets and their parent bodies, possibly leading to the melting of water ice and to the triggering of serpentinization and FTT reactions. Long-lived isotopes such as ^40^K, ^235^U, ^238^U, and ^232^Th, however, should only affect the largest objects during the late stages of their evolution.

### 7.1. Early evolution of comets

**FIG. 4.**
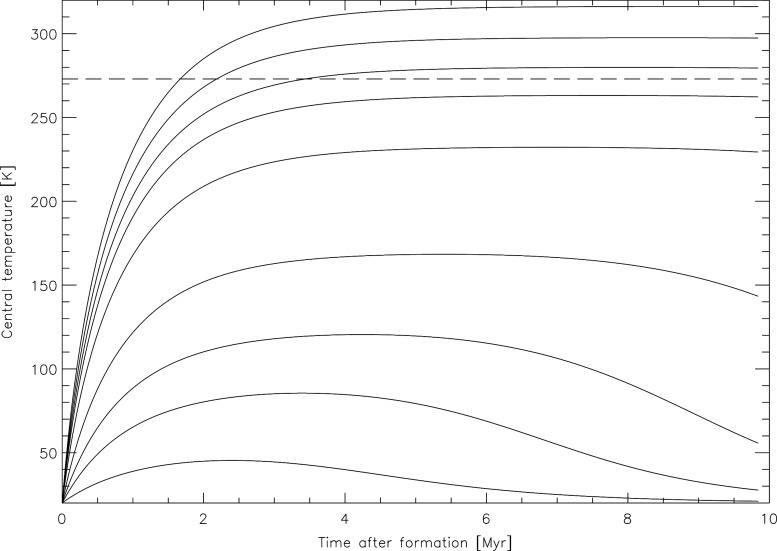
Central temperature of a 2 km radius comet nucleus, under the influence of heating by the radioactive decay of short-lived nuclides ^26^Al and ^60^Fe, as a function of time after formation (no accretional heating is accounted for here). The dashed line highlights the melting point of water. Each solid line represents the evolution of the central temperature after a specific formation time, from 0 (top curve) to 3 million years (bottom curve) since the formation time affects the effective amount of decaying nuclides contained in the body. Thermophysical parameters like thermal conductivity or composition used in the simulations (performed with the model described by Guilbert-Lepoutre *et al.*, 2011) are standard for comets and within the range of realistic values described by other authors.

The effect of radiogenic heating on comets is extremely uncertain, given the number of poorly constrained parameters involved, such as composition, initial internal structure, formation time, radioactive isotope content, porosity, or thermal conductivity. However, having a very short lifetime, the effectiveness of ^26^Al in heating comet interiors strongly depends on the nucleus formation time with respect to formation of calcium-aluminum-rich inclusions. Indeed, Irvine *et al.* (1980), Wallis (1980), and Prialnik *et al.* (1987) showed that, depending on the amount of ^26^Al related to the still poorly constrained comet formation time, the heat produced would be sufficient to melt water ice, in particular, in the case of objects with radii larger than 6 km. Thermal histories are also very sensitive to the material thermal conductivity (Haruyama *et al.*, 1993). Prialnik and Podolak (1995) showed that, depending on the object's size, thermal conductivity, porosity, and initial composition (whether water ice is initially amorphous or crystalline), the early thermal evolution under the influence of ^26^Al decay could lead to various final configurations, ranging from pristine structures being thoroughly preserved to extensive melting of the ice contained in these objects. Here, we show similar results obtained with a model fully described by Guilbert-Lepoutre *et al.* (2011). In this model, the temperature distribution is computed inside an object and at its surface as a function of time and orbital position. For a set of initial thermophysical parameters typical for comets, we illustrate the same effect as Prialnik and Podolak (1995) that different formation times (or equivalent, different initial amounts of ^26^Al) might result in various final structures ranging from fully differentiated to completely pristine (Fig. 4). In summary, specific results on the early heating of comets by radioactive isotopes alone thus require detailed investigations on comet formation processes and timescales, which are not constrained yet, but so far all models are consistent with a potential occurrence of liquid water inside comets for a given set of realistic initial parameters.

It is worth mentioning that the occurrence of high internal temperatures and liquid water is strengthened when accounting for the effect of accretional heating, affecting the early evolution of comets concurrently with radiogenic heating during the short lifetime of ^26^Al. The two processes were combined in a single model by Merk and Prialnik (2006), who showed that the early occurrence of liquid water in 2–32 km radius bodies may be a very common phenomenon. For example, they found that, for a given set of initial parameters, all accreting objects with a final radius above 4 km could produce liquid water cores, extending from 10–90% of the overall interior. Merk and Prialnik (2006) also found that, for a large region of parameter space, liquid water is a rule rather than an exception and that this phase can last for up to 5 million years. The lifetime of the liquid water phase within such bodies should be long enough to trigger serpentinization reactions and the production of H_2_ in their interiors. Fischer-Tropsch-type reactions could have subsequently led to the formation of CH_4_ and higher-order hydrocarbons in comets from the interaction of produced H_2_ and carbonaceous matter. In this context, hydrocarbons present in comets could come from two distinct reservoirs. A significant fraction of these volatiles would have been directly captured in the building blocks of comets at their formation time in the nebula, and another fraction would have been produced directly in their interiors. The relative sizes of these reservoirs will require dedicated studies to be quantified.

### 7.2. Liquid water in Kuiper Belt Objects

**FIG. 5.**
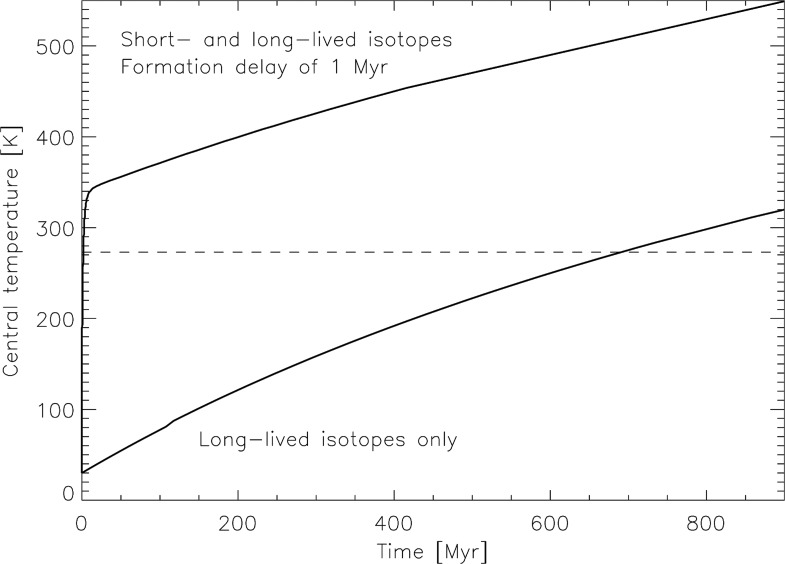
Central temperature of an Orcus-like object (radius of 500 km, density of 1.9 g/cm^3^) as a function of time after formation (no accretional heating is accounted for here). The dashed line highlights the melting point of water, while the solid lines give the evolution of the temperature (computed with the model by Guilbert-Lepoutre *et al.*, 2011) under the influence of long-lived isotopes only (bottom curve) or both short- and long-lived isotopes (top curve, after a formation delay of 1 million years, which decreases the amount of short-lived nuclides). Other parameters of influence, like the thermal conductivity or composition, are within the range of realistic values used by other authors and similar to those used by Delsanti *et al.* (2010).

The thermal evolution of larger objects is more complex. First, it involves two stages: an early evolution, dominated by the radioactive decay of short-lived nuclides ^26^Al and ^60^Fe, which should generate an intense but short heating. Whatever happens during this phase, long-lived isotopes ^40^K, ^235^U, ^238^U, and ^232^Th would inevitably decay over the age of the Solar System, generating a possible more moderate, but more extended, heating of KBO interiors during their late evolution (see the example in Fig. 5). McKinnon *et al.* (2008) found that KBOs with radii larger than 400 km would be the most affected by this late heating. Second, because these objects are larger and denser than comets, the possible production of an internal liquid phase might lead to a significant differentiation of their internal structure, local changes of composition or thermophysical parameters such as thermal conductivity, chemical reactions, or the triggering of processes such as cryovolcanism, which cannot be studied with the same models as those used for small comets. These models would, in this case, be very close to those used by the icy satellite community, and the results could be compared to those found for objects such as Enceladus, for example. Prialnik and Merk (2008) indeed studied the evolution of intermediate-sized objects (250 km radius), including KBOs and icy satellites, accounting for their growth phase and both early and late evolution; in all cases considered, the melting point of water ice was reached.

Including amounts of volatiles that can decrease the melting point of the material, such as NH_3_, CH_4_, or CH_3_OH, has been shown to allow for long-lived liquid phases to be sustained inside the largest KBOs. For example, McKinnon *et al.* (2008) found that, by adding some NH_3_ and CH_3_OH to the water, ice could lead to the production of a liquid phase for all objects with radii larger than 300 km. With water ice alone, the melting point would be reached for all objects with radii larger than 425 km. By including some NH_3_ to water, Hussmann *et al.* (2006) obtained subsurface oceans at the present time in Pluto, Eris (whatever the amount of NH_3_ considered in the models), Sedna, and Orcus. Beyond those theoretical results, KBO observations have recently called for the need to invoke internal liquid phases. Spectral features consistent with the presence of crystalline water, NH_3_, CH_4_, and other volatiles have been detected on spectra of intermediate and large KBOs. Since these features should be erased due to irradiation in short timescales, their presence at the surface of KBOs requires that these compounds were emplaced recently. After eliminating many processes, Cook *et al.* (2007) suggested that cryovolcanism could indeed be the best process to explain the observed surface composition of Charon (crystalline water ice with traces of ammonia hydrates), provided sufficient NH_3_ was incorporated into the object at birth. This was confirmed by the thermal evolution models computed by Desch *et al.* (2009) and by the conclusions of Delsanti *et al.* (2010) to explain the surface composition of Orcus (crystalline water ice with traces of CH_4_, NH_3_, and their irradiation products). These thermodynamic conditions, which are similar to those that occur in the interiors of icy satellites, should then be favorable to serpentinization and FTT reactions and lead to the *in situ* production of hydrocarbons as it is envisaged in Enceladus (see Section 6 for details).

## 8. Conclusions

Serpentinization and the related formation of H_2_ is a favorable and disequilibrium process capable of occurring in a wide variety of celestial bodies. Molecular hydrogen, once formed, has the potential to reduce simple oxidized carbon compounds (*i.e.*, ${ \rm HCO}_3^ {\ -}$, CO, CO_2_) to more reduced species, CH_4_ in particular. Fischer-Tropsch-type processes are critical for H_2_ to react with carbon sources to advance the synthesis of organic compounds. The production of these simple organic compounds provides a feasible pathway related to the origin and sustainment of life. One of the major limitations of serpentinization is the presence and availability of water, which is directly related to the chemical nature, thermal evolution, and size of the celestial body. Provided that water and Fe-bearing silicates such as olivine are present, serpentinization can be invoked to describe the formation and presence of CH_4_ and a wide variety of organic compounds.

## Abbreviations Used

FTT: Fischer-Tropsch-type
KBOs: Kuiper Belt Objects
